# Management of COVID-19 Infection Associated Lung Abscess with Secondary Pleural Empyema Using Percutaneous Intracavitary Drainage: Case Series and Review of the Literature

**DOI:** 10.3390/jcm13226962

**Published:** 2024-11-19

**Authors:** Emanuel Palade, Ioana-Medeea Titu, Angela Elena Goia, Tudor Dan Simu, Sergiu Adrian Ciulic, Simona Manole, Monica Mlesnite

**Affiliations:** 1 Department of Surgery, Iuliu Hatieganu University of Medicine and Pharmacy, 400000 Cluj-Napoca, Romania; paladeemanuel1@gmail.com; 2 Thoracic Surgery Clinic, Leon Daniello Clinical Hospital of Pneumology, 400371 Cluj-Napoca, Romania; 3 Department of Radiology and Medical Imaging, Iuliu Hatieganu University of Medicine and Pharmacy, 400012 Cluj-Napoca, Romania; 4 Department of Radiology, Niculae Stancioiu Heart Institute, Calea Motilor 19-21, 400001 Cluj-Napoca, Romania

**Keywords:** COVID-19, lung abscess, pleural empyema, intracavitary drainage, conservative treatment

## Abstract

**Background/Objectives**: COVID-19-related pulmonary complications, such as lung abscesses and pleural empyema, are rare but serious. This study presents a case series of three patients with COVID-19-associated lung abscesses complicated by pleural empyema, managed conservatively with percutaneous intracavitary drainage (ICD) and lavage. We assess the efficacy and safety of this treatment and compare our findings with the current literature. **Methods**: A retrospective analysis of three cases treated at the Clinic of Thoracic Surgery and Intensive Care Unit in Cluj-Napoca, Romania, was conducted. All patients developed severe lung involvement post-COVID-19, with abscesses rupturing into the pleural cavity. Conservative management included percutaneous ICD and daily lavage with isotonic saline, avoiding extensive surgery due to the patients’ critical condition. Clinical, radiological, and functional outcomes were followed, and results were compared to similar cases in the literature. **Results**: Among 496 critically ill patients with COVID-19 infection, three patients (age 42–60) developed lung abscesses and bacterial superinfection. In all patients, the germs involved were identified (monomicrobial infection in 1, polymicrobial in 2 patients). The abscesses were treated with percutaneous ICD and lavage, leading to clinical improvement in all cases. Long-term drainage (94–290 days) was necessary to obtain healing, and none of the patients required lung resection or decortication. Serial CT scans showed resolution of the abscesses and empyema. All were successfully discharged, and long-term follow-up (30–32 months) revealed minor radiological sequelae and mild respiratory impairment. The literature review found three studies summarizing 45 patients with lung abscesses complicating COVID-19 infections, but only one study addressed the use of percutaneous ICD. The mortality reported in this group was high (50–65%). **Conclusions**: Conservative treatment with percutaneous ICD and lavage is effective in managing lung abscesses and pleural empyema in critically ill COVID-19 patients, offering a viable alternative to surgery in high-risk cases. This method may be beneficial in multidisciplinary care for non-surgical candidates.

## 1. Introduction

Coronavirus disease 2019 (COVID-19) is an infectious disease caused by a new variant of coronavirus (severe acute respiratory syndrome coronavirus 2 or SARS-CoV-2), which affects mainly the respiratory tract and frequently causes viral pneumonia and respiratory insufficiency [1]. About one-third of the patients with SARS-CoV-2 infection requiring hospitalization develop acute respiratory distress syndrome (ARDS) [2]. Several comorbidities, such as hypertension, diabetes, obesity, cardiovascular and kidney diseases, were identified as risk factors for increased mortality [3].

Since the beginning of the COVID-19 pandemic, most countries’ health systems have been overloaded due to the high number of new cases, the rapid spread of the disease, and the high prevalence (about 20%) of severe cases. Several scoring systems have been proposed in order to predict a possible adverse evolution, to reduce the number of severe cases, and to improve outcomes. Quantitative computed tomography lung COVID (QCOVID) scores, based on the analysis of different patterns on high-resolution computer tomography (quantitative ground-glass opacity QGGO, mixed disease QMD, consolidation QCON, normal lung QNL), can be used to calculate the quantitative total lung disease (QTLD = QGGO + QMD + QCON) and offers the radiologists a tool to assess the extent and severity of COVID-19 lung involvement objectively. Quantitative CT COVID scores at admission are able to predict rapid progression of pulmonary lesions, with QTLD score properly assessing COVID-19 pneumonia and QMD score showing the best predictive power for rapid progression. Together with laboratory markers, quantitative CT COVID scores can help clinicians predict the rapid progression of COVID-19 early and make informed decisions [4].

In cases with hypoxemia, refractory to non-invasive treatment options, invasive mechanical ventilation (IMV), or even extracorporeal membrane oxygenation (ECMO) are mandatory. For intubated patients, prolonged mechanical ventilation is needed in up to half of cases, and safe extubation is possible in less than 50% of cases [5]. Additionally, with 45.4%, the incidence of ventilator-associated pneumonia (VAP) in patients with SARS-CoV-2 ARDS is significantly higher than for other causes of ARDS. Of these patients, about 14% develop isolated or multiple lung abscesses, further increasing the disease severity and complicating the treatment, especially as these lesions can open into the pleural cavity, producing pleural empyema [6,7]. Furthermore, COVID-19 patients with ARDS, especially those needing mechanical ventilation, have an increased incidence (5–13%) of pneumomediastinum. This complication occurs after a median of 9 days (IQR 3–13 days) of mechanical ventilation and large airway cartilage lesions, regeneration impairment, and fibrous-hyaline degeneration of tracheal rings as a COVID-19-specific feature was found on autoptic specimens. Two findings, an aberrantly expressed Wnt5a and a weakly expressed SHH (sonic hedgehog) in the injured cartilage tissue, were established in COVID-19 patients with pneumomediastinum compared with non-COVID-ARDS patients. As both represent essential pathways in the repair of cartilage lesions, those abnormalities may eventually explain the high incidence of pneumomediastinum in COVID-19 ARDS patients [8].

Regarding lung abscesses in general, the majority are treated successfully by long-term antibiotics. The emergence of antibiotics reduced the mortality from 75% to about 8.7%. Invasive treatment methods are indicated mainly in cases refractory to antibiotics. As minimally invasive procedures, bronchoscopic endobronchial drainage and percutaneous intracavitary drainage (ICD) are described. Endobronchial drainage is rarely used due to the risk of bacterial contamination of unaffected lung areas, and it is recommended mainly in patients with poor general conditions, coagulopathies, and centrally located abscesses. Percutaneous ICD, first described in 1938 for cavitary tuberculosis, is a more feasible method. It was routinely adopted in the pre-antibiotic era for lung abscess treatment and is currently being used in about 11–21% of cases refractory to antibiotics. Nowadays, ICDs are placed using ultrasound or CT guidance with acceptable drainage-related morbidity of 16% and mortality of about 4%. Complications include bleeding, bronchopleural fistula, and pyopneumothorax. Major surgery represented by anatomic lung resections (mainly lobectomy) is used in about 10% of lung abscesses. Common indications consist of abscess persistence of more than 6 weeks, suspicion of cancer, cavities larger than 6 cm, hemoptysis, sepsis refractory to antibiotics, and bronchopleural fistula with empyema. In rare cases, open abscess drainage (cavernostomy) may represent a surgical treatment option [9].

The aim of this study is to summarize the management of a rare complication of COVID-19 pulmonary involvement represented by bacterial superinfection with abscess formation, based on our experience with conservative treatment of lung abscesses complicated with pleural empyema using percutaneous intracavitary drainage (ICD) and lavage. As a novelty, we offered a detailed presentation of our standardized approach with percutaneous ICD as a valuable treatment option in severely compromised patients that are no candidates for more aggressive surgery.

## 2. Materials and Methods

We analyzed the collected data and results and compared them with the results of other studies, offering a review of the actual literature on this topic.

The study is a retrospective single-center analysis of the records from the Department of Thoracic Surgery and Intensive Care Unit during the COVID-19 pandemic, presenting the conservative treatment of 3 patients with superinfection of SARS-CoV-2 pneumonia, complicated with lung abscess and pleural empyema due to abscess rupture into the pleural cavity. We describe in detail our concept of conservative treatment (intracavitary drainage and intermittent lavage) used in these cases, a treatment method we also use frequently in other patients with lung abscesses (with or without pleural empyema), which are not candidates for extensive surgery (lung resection, decortication). We also present the evolution, outcome, and particulars of the cases.

**Figure 1:**
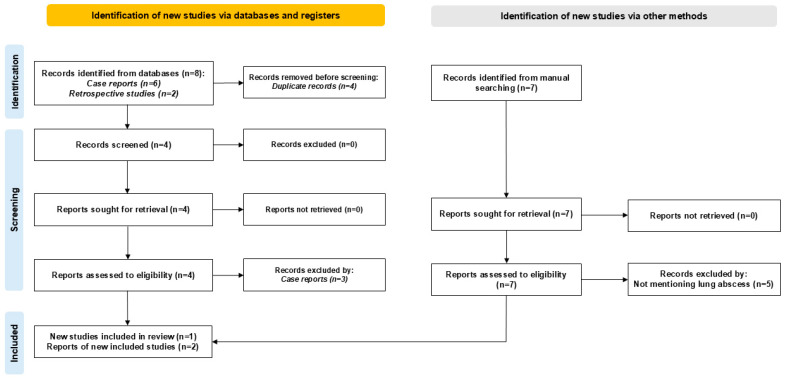
PRISMA diagram showing the results of the literature search.

A systematic literature search was conducted in accordance with the Preferred Reporting Items for Systematic Reviews and Meta-Analyses (PRISMA) guidelines to ensure methodological rigor, transparency, and reproducibility throughout the review process. The search included articles published in the PubMed database from January 2020 to the present, with a supplementary manual search undertaken to identify any additional pertinent studies potentially omitted in the primary database search. The grey literature, including theses, dissertations, and conference proceedings, was excluded from this review to prioritize peer-reviewed, published studies. Figure 1 shows the search results using the PRISMA diagram.

To enhance the specificity of the search, medical subject headings (MeSH) were employed to identify studies explicitly addressing lung abscesses in the context of COVID-19. The search strategy applied the following terms: “COVID-19” (Mesh) AND “Lung Abscess”(Mesh) AND “SARS-CoV-2”(Mesh) AND “Lung Abscess”(Mesh). These terms were strategically chosen to facilitate the retrieval of studies that investigate the incidence, clinical manifestations, pathophysiology, therapeutic approaches, and outcomes of lung abscesses associated with COVID-19.

## 3. Results

Among the 496 critically ill patients treated on ICU, 169 required mechanical ventilation, and 3 were identified with lung abscesses. Of all patients treated in the ICU, 101 succumbed to the disease.

Of the three cases, two were females and one male, aged between 42 and 60 years. All three were not vaccinated against COVID-19, and in terms of associated comorbidities, one had hypertension, one had obesity, and the third one had a combination of both comorbidities (multimorbidity). A typical COVID-19 patient clinical presentation was tested positive using RT-PCR (nasopharyngeal swab). Table 1 presents the patients’ characteristics and summarizes the radiologic and clinical evolution, treatment used, and outcome.

**Figure 2:**
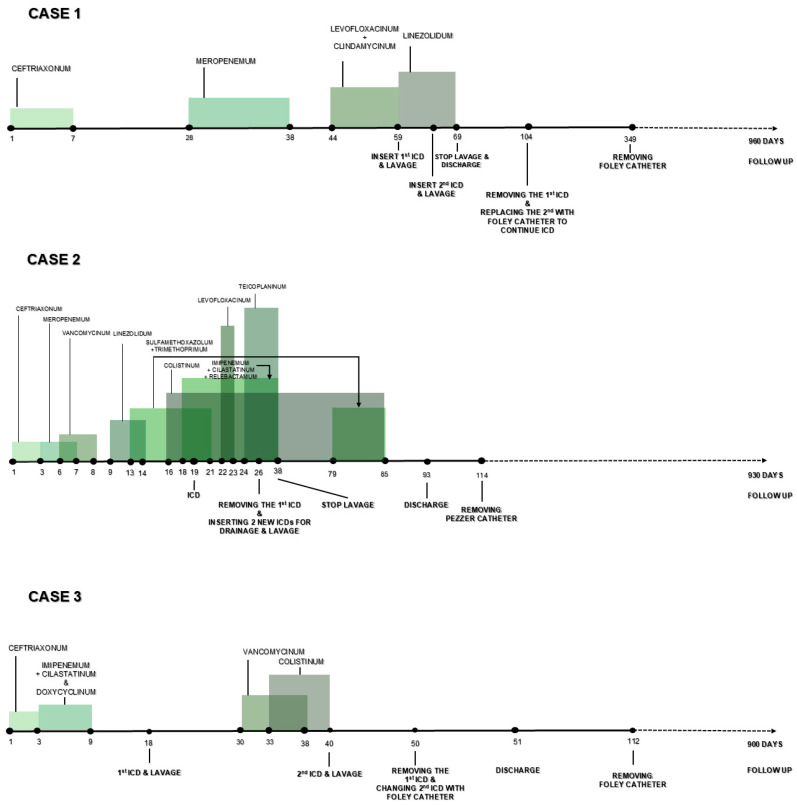
Antibiotic treatment used, ICD and lavage duration, discharge, and follow-up in all three cases.

Despite the prompt initiation of therapy (antiviral, oxygen supplementation, antibiotics), all three patients developed significant pulmonary involvement (75% in case 1, over 90% in cases 2 and 3), necessitating various types of respiratory support from non-invasive ventilation to invasive mechanical ventilation and eventually veno-venous extracorporeal membrane oxygenation (VV-ECMO) therapy in one case. Figure 2 summarizes the antibiotic treatment used, ICD and lavage duration, discharge, and follow-up.

As a result of the extended hospitalization period, poor functional status, and different antibiotic regimes, the patients developed COVID-19-associated multidrug-resistant pneumonia, further complicated by lung abscesses and finally with pleural empyema.

**Figure 3:**
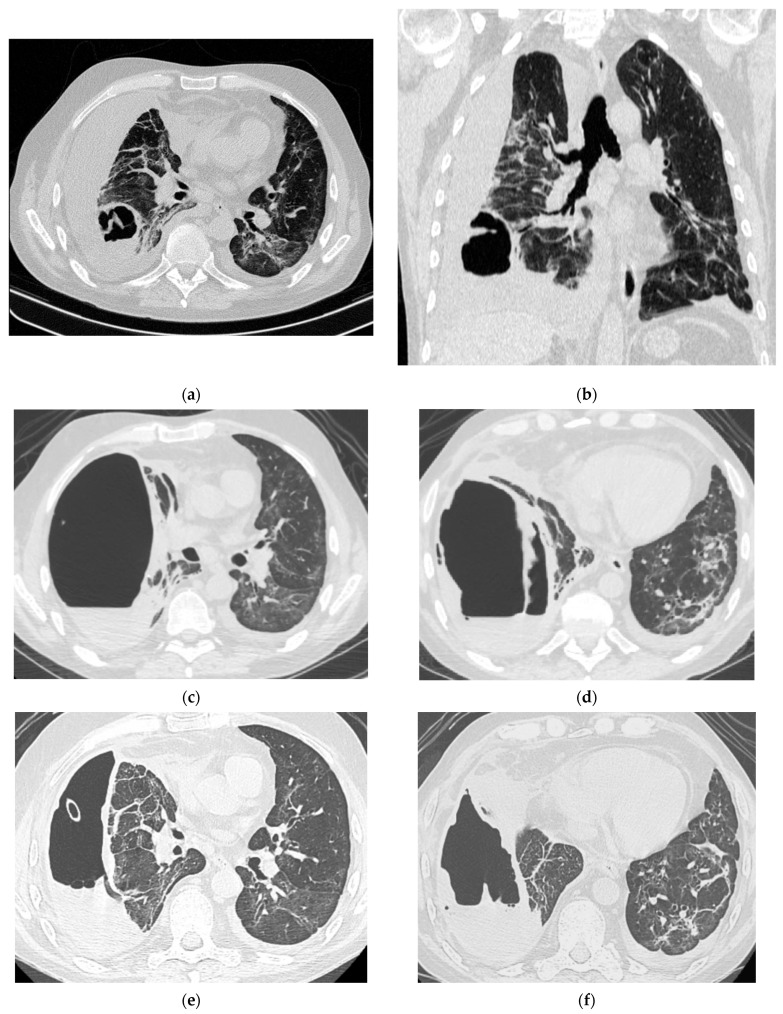

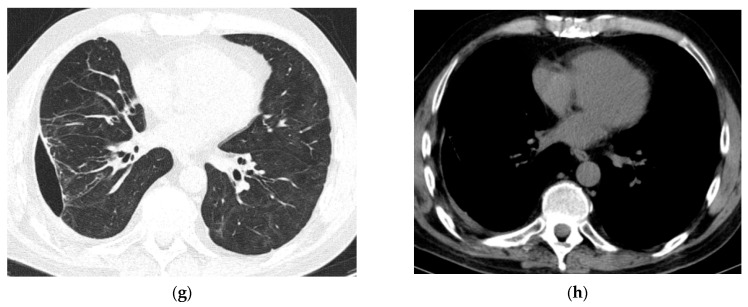
Radiologic evolution of case 1: Initial chest CT scan (axial and coronal plane) showing right lower lobe abscess prior to rupture into pleural space and post-COVID-19 infection infiltrates (**a**,**b**). CT scan after abscess rupture and prior to percutaneous drainage showing air cavity with dense fluid and hydro-aeric level occupying almost the entire right lung field; remaining right lung parenchyma with fibrotic changes, and a large lung abscess compressed at the hilum; small left pleural collection; small pericardial effusion (**c**,**d**). After the insertion of percutaneous ICD, CT scans follow-up reveals decreased volume of the residual cavity, thickened pleura and intracavitary drainage (**e**); slightly re-expanded right lung parenchyma; numerous ground-glass opacities (GGO), areas of fibrosis and passive atelectasis (**e**,**f**). After chest tube removal (290 days of drainage), minimal residual cavity without fluid and thin pleural fibrotic changes remained (**g**,**h**).

**Figure 4:**
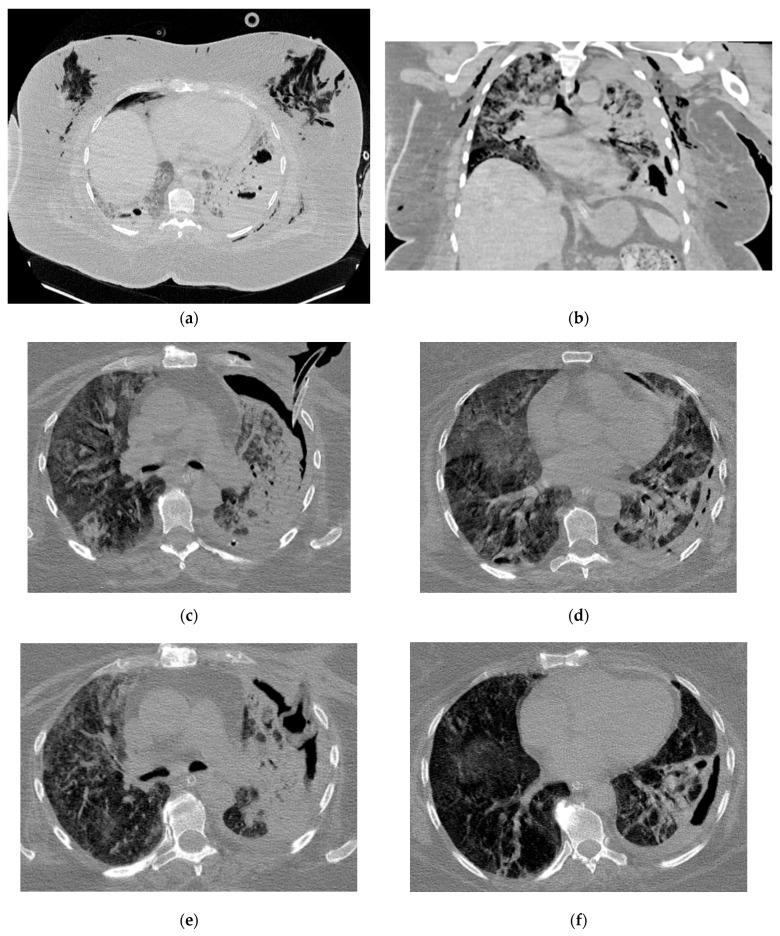

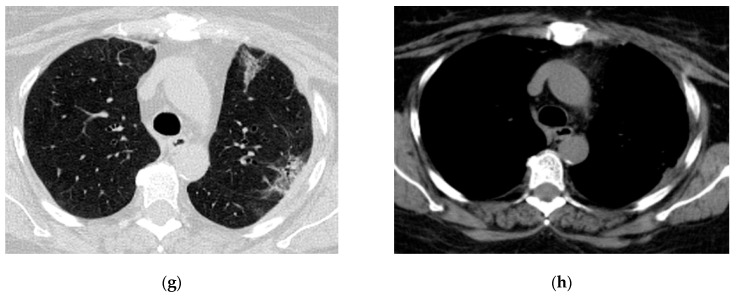
Radiologic evolution of case 2: Initial axial and coronal chest CT scans with left lower lobe abscess prior to rupture into the pleural cavity, extended bilateral lung infiltrates, and consolidations (**a**,**b**). Inserted percutaneous ICD used for both pleural lavage and drainage, trapped lung with small fluid retention (**a**), diffuse pulmonary infiltrates in both lung fields (**c**,**d**), and small left pulmonary cavitary lesions (**d**). Chest CT scans follow-up reveals intracavitary Pezzer catheter (used instead of Foley catheter due to a large soft tissue defect secondary to chest tube infection) and left-sided pyopneumothorax with mixed density fluid after lavage, ground-glass opacities (GGO) and left pulmonary consolidations areas (**e**,**f**). Six months follow-up shows complete closure of pleural and pulmonary cavities, and resolution of pulmonary infiltrates except for two small fibrotic areas (**g**,**h**).

**Figure 5:**
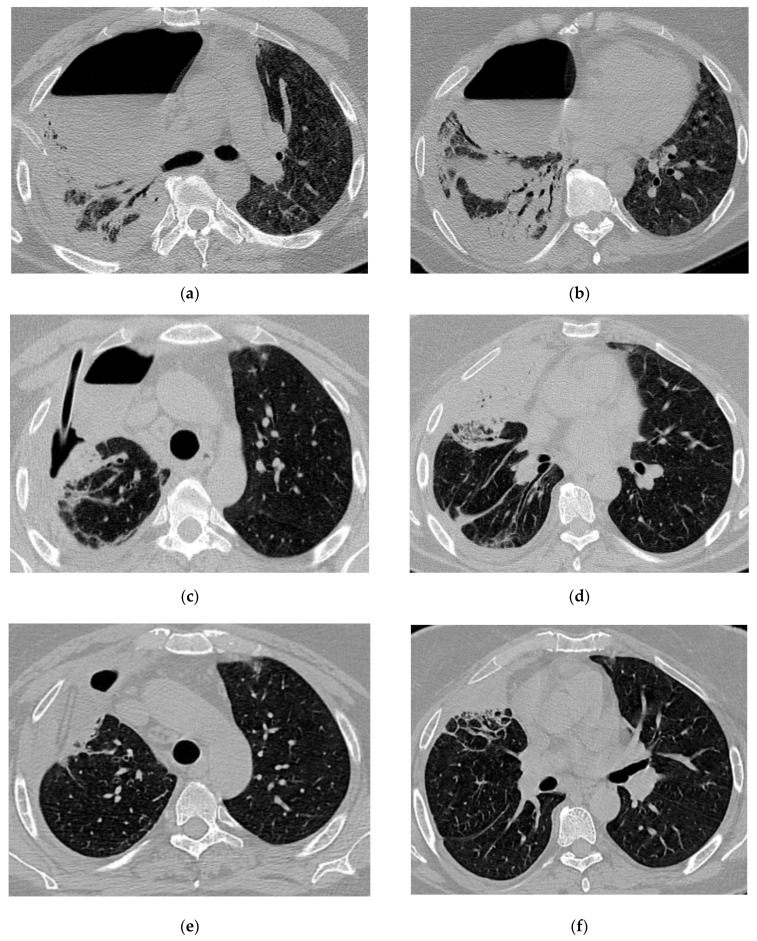
Radiologic evolution of case 3: Axial chest CT scans after first percutaneous ICD insertion revealing insufficient drainage of the purulent cavity; pleural effusion with few air inclusions; right ICD and diffuse interstitial infiltrates (**a**,**b**). In this case, multiple drainage and drain revisions were necessary to optimally control the purulent infection. Smaller right residual cavity with stable pleural effusion after ICD revision (**c**,**d**). Chest CT scans 1-month follow-up show the Foley catheter and decreasing size of the residual cavity (**e**). A follow-up CT scan after ICD removal showed small residual pleural and pulmonary fibrotic changes (**f**).

Pneumologists and intensive care specialists treated the patients until the lung abscesses were complicated with pleural empyema, and thoracic surgeons were involved in the treatment. Due to the severity of the COVID-19 pulmonary damage and the severe respiratory insufficiency, major surgery (lung resection, decortication of the lung) was contraindicated, and the therapeutic management of all cases was conservative. A percutaneous intracavitary drainage (ICD) was inserted, and lavage of the pleural cavity with isotonic saline solution through the inserted drainage was initiated as soon as the bronchopleural fistula was no longer manifest. In some cases, multiple chest tube insertions were mandatory to reach the entire empyema cavity. In all cases, after initiation of this treatment, the clinical course gradually improved due to the rapid control of the purulent infection. Parallel to that, radiologic regression of the pulmonary infiltrates and gradual functional recovery were noticed. The evolution of the purulent cavity (lung abscess and pleural empyema) was documented by serial CT scans, and the local and systemic treatment was adapted as necessary. The outcome of all patients was favorable, finally allowing the suppression of the ICD. Figure 3, Figure 4 and Figure 5 showcase the evolution of purulent infection under the conservative treatment, offering insight into this treatment method.

In our clinic, conservative treatment using percutaneous ICD and lavage is extensively used with very good results in all patients with pleural empyema, lung abscesses, or a combination of both lesions, which are no candidates for extended surgery (lung resection, decortication) due to their poor condition or comorbidities. The strategy implies in cases of lung abscess without pleural empyema to drain the purulent collection by CT-guided insertion of a percutaneous intracavitary catheter (in order to reduce the risk of complications such as lung injury, bleeding, and rupture in the pleural space) or to insert a chest tube (20–24 CH) in the purulent cavity in cases with lung abscess and pleural empyema due to abscess rupture in the pleural space. Additionally, in cases without bronchopleural fistula, daily intermittent lavage of the purulent cavity using 500 mL isotonic saline solution until the lavage fluid turns serous is performed. We do not add disinfectants in the saline solution, and the duration of lavage is, in general, 5 to 10 days. The positive role of lavage consists in aiding the evacuation of purulent and necrotic debris, reducing the number of bacteria, and accelerating the healing process. Another particularity in the drainage therapy we use is that in cases with good results (assessed by clinical and radiological improvement) of the treatment, after approximately 10 days, we replace the chest tube with a large bore (20–22 CH) Foley catheter. This brings, in our opinion, several advantages: location at the lowest point of the infected cavity, very well tolerated in terms of reduced pain and inflammation at the insertion point, and no need for fixation. At some point, the patients can eventually be discharged and treated in an outpatient setting until the drainage is removed. The healing of the abscess cavity has to be documented by a CT scan. This chronic drainage treatment has the advantage of very low invasivity and, therefore, is extremely useful in patients at high risk for more invasive surgical procedures, especially when the purulent cavity can be effectively drained. The disadvantages include prolonged treatment time (weeks, even months), repeated clinic and CT scan presentations, and persistence of various degrees of fibrothorax. With the exception of case 2, in which a minor complication (wound infection at the drainage site treated locally, without removing the drainage) was encountered, no further complications were found.

## 4. Discussion

Lung abscesses consists of cavities more than 2 cm in diameter located in the lung parenchyma, filled with necrotic debris or purulent fluid. They are caused by microbial infections and can be primary (60% of cases) complicating bacterial pneumonia or secondary to superinfection of preexisting lung lesions (cysts, emphysema bullae, pulmonary infarction, etc.) or caused by hematogenous spread in the lung from extrapulmonary infections (e.g., liver abscess). The majority of lung abscesses are caused by aspiration from the oral cavity; VAP is also an important etiologic factor. Lung abscess treatment consists mainly of antibiotic therapy to which endobronchial drainage, percutaneous intracavitary drainage (ICD), or lung resection can be added depending on the extent of the disease, severity, and evolution [9]. In some cases, pleural empyema can further complicate lung abscesses, with or without bronchopleural fistula, by rupturing into the pleural space and making thoracic surgical treatment mandatory.

Percutaneous drainage of lung abscesses is a well-known treatment option, but it has been restrictively used in the past due to concerns related to possible complications [10]. As pointed out by a recent meta-analysis including a total of 832 patients (412 in the control group treated with antibiotics and 420 in the intervention group treated with antibiotic and percutaneous drainage) from 13 trials (10 randomized and 3 non-randomized) published between 2010–2019, the intracavitary catheter drainage additionally to antibiotics improves the treatment outcome in terms of effectivity rate (*p* < 0.01), shorten of hospital stay and number of fever days (*p* < 0.01) without significant difference in complication rate (*p* = 0.43) compared with antibiotic treatment without ICD [10].

In COVID-19 pulmonary involvement, bacterial superinfection occurs in 7–14% of cases, which is relatively rare compared with superinfections in influenza (11–35%) [11,12]. The incidence of bacterial superinfection is higher in hospitalized patients (12%) than in those treated ambulatory (6%) [13]. On the other hand, the incidence of VAP seems, with 47–73%, to be significantly higher than in other causes of ARDS [7,14]. In our department, the incidence of VAP was 14.8%.

As for other types of pneumonia, superinfection of COVID-19 pulmonary lesions can lead to lung abscesses, especially when VAP occurs. The review of the literature revealed only three studies (two single-center and one multi-center) addressing the incidence and treatment of lung abscesses in COVID-19 infection and only one (a single-center study) of those discussing the use of percutaneous drainage of the purulent cavity [7,14,15]. Additionally, several single case reports were published that were not included in our analysis.

In one single-center study (Beaucoté et al., 2021) from 119 COVID-19 patients with VAP, 17 patients (14%) developed lung abscesses, all treated with antibiotics without ICD or other surgical procedures and showing a mortality rate of 65%, not significantly higher (*p* = 0.57) as for VAP without lung abscess (54%) [7]. In the multi-center study from Hraiech et al. [15], from a total of 507 patients with IMV from 3 ICU’s, 23 (7%) developed a lung abscess. Although 12 patients developed surgical complications (4 pleural empyema and 8 pneumothorax), from which 7 (30%) were treated with thoracic drainage or decortication, the lung abscess treatment was based on antibiotics without ICD or lung resection. Further analysis of the patients receiving thoracic drainage was not offered. The mortality of patients with VAP and lung abscess was 52%, not significantly higher than for patients with VAP without lung abscess (35%) [15]. The highest incidence of lung abscess complicating VAP in COVID-19 patients (about 17%, *n* = 5) was found in the single-center retrospective analysis published by Shu Utsumi et al. in 2023. A total of 6 patients (20%) out of 30 with VAP developed lung abscess (4 patients), lung abscess and pleural empyema (1 patient) or pleural empyema alone (1 patient). All lung abscesses occurred in the right lung (three upper lobes, two lower lobes) and were treated with antibiotics and percutaneous drainage for the two cases with pleural empyema and antibiotics without ICD for those with lung abscess alone. Although details about the drainage therapy (use of lavage, length of drainage) were not offered, these complications occurred within a median time of 15 days (IQR, 10–18) after tracheal intubation and a median time of 4 (2–7) days after the onset of VAP. A higher mortality (50 vs. 25%) tended (*p* = 0.33) to occur in the group with complications as in the group of non-complicated VAP [14].

In comparison with these data, in our study, all patients survived, probably also due to the meticulous surgical treatment of the purulent infection. Another important aspect is that in our group, only one patient had VAP; in the other two cases, pneumonia complicated with abscess and pleural empyema was not ventilator-associated as the patients received non-invasive ventilation (CPAP) in one case, and awake veno-venous ECMO in the other case. In all our cases, lung abscesses occurred as a complication of bacterial superinfection of COVID-19 pulmonary lesions, in one case based on VAP and in two cases without invasive mechanical ventilation (IMV). Predisposing factors, such as pulmonary embolism (one case) or pulmonary vascular endothelialitis with thrombosis frequently found in critically ill COVID-19 patients, may have facilitated the development of lung abscesses. One possible mechanism may be the impaired penetration of antibiotics in the superinfection area, as suggested by Beaucoté et al. The same author found an incidence of 18% of pulmonary embolism or thrombosis in their single-center analysis of 17 lung abscesses out of 119 VAP COVID-19 patients [7].

Regarding the microbial flora involved, in our study, two out of three cases (67%) had a polymicrobial infection. Similar findings, with 80% and 65% polymicrobial flora, were also reported by other authors. Enterobacteriaceae, Pseudomonas aeruginosa and Staphylococcus aureus were most frequently identified, but Acinetobacter species and Stenotrophomonas maltophilia were also encountered [7,15]. Although we report on only three cases, the microbiologic findings are very similar to those from larger series. Unlike these studies, Shu Utsumi et al. found only monomicrobial infections, with Staphylococcus aureus in four cases and Klebsiella species in the remaining two. The authors attribute this finding to the restrictive use of antibiotics in their ICU [14]. Most interestingly, in all these studies, the microbiologic yield from endobronchial secretions was very high, up to 100%, making abscess punction unnecessary.

The duration of treatment varied among patients. For case 1, inpatient drainage lasted 10 days, with outpatient treatment continuing for up to 280 days. In case 2, the patient underwent drainage in the hospital for 74 days, with the drainage being discontinued on the 95th day in an outpatient setting. In case 3, the patient received inpatient drainage for 43 days, followed by 51 days of drainage in an outpatient setting prior to removal. They subsequently had a clinical and radiological follow-up period of 32, 31, and 30 months. To our knowledge, our study is the only one offering detailed information about ICD strategy and treatment timeline.

Although the analyzed studies on lung abscesses in COVID-19 patients do not refer to abscess healing (with or without sequelae) in all our cases, discrete radiologic sequelae (small pleural cavities without fluid, localized pleural thickening, small pulmonary infiltrates) were present at the end of the radiologic follow-up. As for functional impairments, only mild respiratory impairment was assessed in all cases.

As noted by Beaucoté et al., predictive factors for the development of lung abscesses in COVID-19 patients couldn’t be identified as the proportion of patients with debilitating conditions such as diabetes mellitus, chronic respiratory disease, bacterial coinfection at ICU admission or immunosuppression including corticosteroid therapy were not different to patients without lung abscess [7]. Therefore, the issue of early detection of lung abscesses in order to limit their evolution by adapting the treatment (change in antibiotic therapy, insert intracavitary or endobronchial drainage) and prevent further complications such as pleural empyema, extension to other lung areas, or hemoptysis, becomes essential.

To our knowledge, this study, even if analysis only a small number (*n* = 3) of patients, represents the largest series of patients with this rare and severe complication of lung abscess opened in the pleural space in patients with COVID-19 infection. Noteworthy is that none of the patients had a lethal outcome, which, in our opinion, can be attributed mainly to the structured concept of ICD with the lavage we used.

The main limitation of the current analysis is the small number of patients, which can probably be improved by performing a multi-center study. Further, a comparison of the treatment used with other surgical options (lung resection, decortication) is impossible due to the extremely high operative risk in these severely compromised patients.

## 5. Conclusions

The present study proved that conservative treatment based on percutaneous ICD and lavage is both effective and safe for lung abscesses and pleural empyema in cases with COVID-19 infection. Especially useful is this treatment option in severely compromised patients who are no candidates for extensive surgery, not only in COVID-19 patients but in general in critically ill patients with lung abscesses and/or pleural empyema. The interdisciplinary treatment with the involvement of a thoracic surgical team and a structured therapy concept of ICD and lavage meticulously implemented are mandatory for good results. As a novelty, detailed information about our percutaneous ICD strategy can help thoracic surgeons, intensive care specialists, and pneumologists effectively address severely compromised patients with lung abscesses and/or pleural empyema.

## Figures and Tables

**Table 1:** Characteristics of the patients, treatment used, evolution, and outcome.

Case	Case 1	Case 2	Case 3
Sex	Male	Female	Female
Age	60	51	42
COVID-19 vaccine	No	No	No
Comorbidities	Hypertension	Obesity (BMI 37.72) Hypertension	Obesity (BMI 35.38)
Clinical medical history	Pulmonary tuberculosis	Uterine fibroid	-
Positive for COVID-19	February 2021	October 2021	December 2021
Treatment	CPAP Antibiotics Corticosteroids Remdesivir Tocilizumab	Invasive mechanical ventilation (32 days) Antibiotics Corticosteroids Favipiravir, Remdesivir Tocilizumab	VV-ECMO (22 days) CPAP Antibiotics Corticosteroids Favipiravir, Remdesivir
Drainage microbiology	*Corynebacterium* spp.	*Pseudomonas aeruginosa* *Stenotrophomonas maltophilia*	*Enterococcus faecalis* *Acinetobacter baumanii* complex
Maximal extent of pulmonary involvement	75%	>90%	95%
COVID-19 complications	Bilateral pulmonary thromboembolism Right lower lobe pulmonary infarction Right lower lobe abscess Right pyopneumothorax	Left upper lobe abscess Left pyopneumothorax	Right upper lobe abscess Right pyopneumothorax
Thoracic surgery management	Chest tube drainage Foley catheter drainage Pleural lavage with isotonic saline solution	Chest tube drainage Pezzer catheter drainage Pleural lavage with Colistin solution	Chest tube drainage Foley catheter drainage Pleural lavage with isotonic saline solution
ICD-related complications	No	Chest tube wound infection (conservative treatment)	No
ICD duration	290 days (10 d inpatient + 280 d outpatient)	95 days (74 d inpatient + 21 d outpatient)	94 days (43 d inpatient + 51 d outpatient)
Follow-up	32 months	31 months	30 months
Actual status	Minimally radiologic sequelae Mild respiratory impairment	Minimally radiologic sequelae Mild respiratory impairment	Minimally radiologic sequelae Mild respiratory impairment

## Data Availability

The data supporting the findings of this study are available from the corresponding author upon reasonable request. Due to ethical considerations, the data are not publicly accessible.
